# *Mkk4* and *Mkk7* are important for retinal development and axonal injury-induced retinal ganglion cell death

**DOI:** 10.1038/s41419-018-1079-7

**Published:** 2018-10-26

**Authors:** Stephanie B. Syc-Mazurek, Rebecca L. Rausch, Kimberly A. Fernandes, Michael P. Wilson, Richard T. Libby

**Affiliations:** 1 Department of Ophthalmology, University of Rochester Medical Center, Rochester, NY USA; 2 Neuroscience Graduate Program, University of Rochester Medical Center, Rochester, NY USA; 3 Department of Biomedical Genetics, University of Rochester Medical Center, Rochester, NY USA; 4 The Center for Visual Sciences, University of Rochester, Rochester, NY USA

## Abstract

The mitogen-activated protein kinase (MAPK) pathway has been shown to be involved in both neurodevelopment and neurodegeneration. c-Jun N-terminal kinase (JNK), a MAPK important in retinal development and after optic nerve crush injury, is regulated by two upstream kinases: MKK4 and MKK7. The specific requirements of MKK4 and MKK7 in retinal development and retinal ganglion cell (RGC) death after axonal injury, however, are currently undefined. Optic nerve injury is an important insult in many neurologic conditions including traumatic, ischemic, inflammatory, and glaucomatous optic neuropathies. Mice deficient in *Mkk4*, *Mkk7*, and both *Mkk4* and *Mkk7* were generated. Immunohistochemistry was used to study the distribution and structure of retinal cell types and to assess RGC survival after optic nerve injury (mechanical controlled optic nerve crush (CONC)). Adult *Mkk4*- and *Mkk7-*deficient retinas had all retinal cell types, and with the exception of small areas of disrupted photoreceptor lamination in *Mkk4*-deficient mice, the retinas of both mutants were grossly normal. Deficiency of *Mkk4* or *Mkk7* reduced JNK signaling in RGCs after axonal injury and resulted in a significantly greater percentage of surviving RGCs 35 days after CONC as compared to wild-type controls (*Mkk4*: 51.5%, *Mkk7:* 29.1%, WT: 15.2%; *p* < 0.001). Combined deficiency of *Mkk4* and *Mkk7* caused failure of optic nerve formation, irregular retinal axonal trajectories, disruption of retinal lamination, clumping of RGC bodies, and dendritic fasciculation of dopaminergic amacrine cells. These results suggest that MKK4 and MKK7 may serve redundant and unique roles in molecular signaling important for retinal development and injury response following axonal insult.

## Introduction

The mitogen-activated protein kinase (MAPK) pathway is involved in development, neurodegeneration, and the immune response^1–5^. In the retina, MAPK signaling plays a role in retinal formation and axonal injury-induced retinal ganglion cell (RGC) death^6–12^. The MAPK, c-Jun N-terminal kinase (JNK), is regulated by two upstream MAP2Ks: MKK4 and MKK7^5,13,14^. The specific requirements of MKK4 and MKK7 in retinal development and neurodegeneration, however, are currently undefined.

MKK4 and MKK7 are required for normal development^15^. In the central nervous system, MKK4 and MKK7 and their downstream effector molecules, the JNKs (JNK1–3), play important roles in both development and maintenance of neural structures. MKK4, MKK7, and the JNKs contribute to the regulation of cellular organization and axonal migration through both overlapping and non-redundant mechanisms^16–19,20^. JNK signaling has also been shown to contribute to multiple aspects of retinogenesis such as progenitor cell proliferation^14,21^. The exact contributions of MKK4 and MKK7 to retinal development, however, remain largely unexplored. In the adult, multiple MAPK members have been shown to be key mediators of the apoptotic injury response and RGC death after axonal injury. Specifically, JNKs and their canonical downstream effector molecule, the transcription factor JUN, are important for RGC death after mechanical- and ocular hypertension-induced axonal injury^7,8,21–24^. Despite this known involvement, the critical molecular events leading from axonal injury to RGC death are not fully defined. Determining the molecular mechanisms of RGC pro-death signaling after axonal injury is necessary for understanding the molecular underpinnings of diseases such as glaucoma and traumatic optic neuropathies which result in RGC loss.

The importance of JNK signaling for both RGC development and response to axonal injury is well established, but little is known regarding the role of the MAP2Ks upstream of JNK in these processes. Selectively targeting these upstream MAPKs may allow us to define the specific pathological signaling pathway that activates pro-death JNK activation in RGC axons after an insult. Furthermore, understanding the contribution of MKK4 and MKK7 to the injury response and to JUN activation in RGCs will likely have implications for other diseases or traumas involving axonal injury. Here, using conditional null alleles of *Mkk4* and *Mkk7*, we demonstrate their redundant and unique roles in retinal development and injury signaling.

## Materials and methods

### Mice

All experiments were conducted in adherence with the Association for Research in Vision and Ophthalmology’s statement on the use of animals in ophthalmic and vision research and were approved by the University of Rochester’s University Committee on Animal Resources. Animals were housed on a 12-h light and dark cycle and received chow and water ad libitum. Floxed alleles of *Mkk4* and *Mkk7*^25,26^ were maintained on the C57BL/6J background. To generate animals deficient in *Mkk4* or *Mkk7*, mice carrying the respective floxed allele were bred to animals carrying *Six3*cre which is first expressed in the optic cup between E9.0 and 9.5^27^. Animals carrying the Cre recombinase and one *Mkk4* or *Mkk7* floxed allele were intercrossed to generate animals: (1) carrying Cre recombinase and two copies of either *Mkk4* or *Mkk7* floxed alleles, referred to as *Mkk4* deficient (*Mkk4*^*−/−*^ or *Six3*cre^+^*; Mkk4*^*f/f*^) and *Mkk7* deficient (*Mkk7*^*−/−*^ or *Six3*cre^+^*; Mkk7*^*f/f*^) respectively and (2) animals without floxed alleles and/or without Cre recombinase, referred to as wild-type controls (*Six3*cre^*+*^; *Mkk4*^*+/+*^, *Six3*cre^*−*^*; Mkk4*^*+/+*^, *Six3*cre^*−*^; *Mkk4*^*f/+*^*, Six3*cre^*−*^; *Mkk4*^*f/f*^, *Six3*cre^*+*^; *Mkk7*^*+/+*^, *Six3*cre^*−*^; *Mkk7*^*+/+*^*, Six3*cre^*−*^; *Mkk7*^*f/+*^, or *Six3*cre^*−*^; Mkk7^*f/f*^). Animals deficient in both *Mkk4* and *Mkk7* were generated by breeding animals carrying the floxed alleles and *Six3*cre together to generate *Six3*cre^+^; *Mkk4*^*f/f*^; *Mkk7*^*f/f*^ animals (*Mkk4/Mkk7-*deficient animals).

### Retinal histology, immunohistochemistry, and cell counts

Eyes used to evaluate retinal morphology were processed in 2.5% glutaraldehyde and 2% paraformaldehyde (PFA) for 24 h at 4 °C. Eyes were then dehydrated with a series of ethanol washes, embedded in Technovit (Electron Microscopy Services), sectioned at 2.5 μm, and stained with Multiple Stain Solution (Polysciences, Inc). Eyes to be processed for immunohistochemistry and retinal flat mounts were harvested and processed in 4% PFA for 2 h at room temperature prior to storage in 1 M phosphate-buffered saline (PBS). The anterior segment of the eye was dissected away and the posterior segment of the eye was processed for cyrosectioning (14 μm sections) or whole retina flat mounts as previously described^7^. For optic nerve morphological analysis, 8 additional animals per genotype were perfused with 4% PFA. Brains were then dissected free and the optic nerves were photographed using a stereomicroscope.

For immunohistochemical staining on cryosections, sections were blocked in 10% horse serum with 0.1% Triton X in 1× PBS for 3 h at room temperature and then incubated in primary antibody overnight at 4 °C. Primary antibodies included: goat anti-SOX2 (1:250, Santa Cruz), goat anti-CHaT (1:200, Millipore), rabbit anti-calretinin (1:1000, Millipore), mouse anti-calbindin-D-28K (1:1000, Sigma), rabbit anti-PKCα (1:2000, Sigma), and rabbit anti-pJNK (1:250, Cell Signaling). Cryosections were washed in PBS and incubated in Alexafluor-conjugated secondary antibodies (Invitrogen) for 2 h at room temperature. Cyrosections were then washed, counterstained with 4′,6-diamidino-2-phenylindole (DAPI), and mounted in Fluorogel in TRIS buffer (Electron Microscopy Sciences). A complete list of antibodies used is provided in Table 1.

**Table 1:** Summary of primary antibodies

	Primary antibody (catalog no.)	Antibody concentration	Company
Immunohistochemistry			
BRN3b	Polyclonal goat anti-BRN3b (sc-6026)	1:250	Santa Cruz
Calbindin	Monoclonal mouse anti-calbindin-D-28K (C9848)	1:1000	Sigma
Calretinin	Polyclonal rabbit anti-calretinin (AB5054)	1:1000	Millipore
cCASP3	Polyclonal rabbit anti-cCASP3 (AB3623)	1:1000	Millipore
CHaT	Polyclonal goat anti-CHaT (AB144P)	1:200	Millipore
NF	Monoclonal rabbit anti-NF (C28E10)	1:100	Cell Signaling
pJNK	Monoclonal rabbit anti-pJNK (4668S)	1:250	Cell Signaling
pJUN	Monoconal rabbit anti-pJUN (D47G9)	1:250	Cell Signaling
PKCα	Polyclonal rabbit anti-PKCα (P4334)	1:2000	Sigma
SOX2	Polyclonal goat anti-SOX2 (sc-17320)	1:250	Santa Cruz
TH	Polyclonal rabbit anti-TH (AB152)	1:1000	Millipore
TUJ-1	Monoclonal mouse anti-TUJ-1 (801202)	1:1000	BioLegend
Western blot			
GAPDH	Monoconal rabbit anti-GADPH (14C10)	1:2000	Cell Signaling
MKK4	Polyclonal rabbit anti-MKK4 (9152)	1:500	Cell Signaling
MKK7	Polyclonl rabbit anti-MKK7 (4172)	1:500	Cell Signaling
pJNK	Monoclonal rabbit anti-pJNK (4668)	1:500	Cell Signaling
pJUN	Polyclonal rabbit anti-pJUN (9261)	1:500	Cell Signaling

For immunohistochemistry on flat mounts, floating retinas were blocked in 10% horse serum with 0.3% Triton X in 1× PBS overnight on a shaker at 4 °C and then incubated in primary antibody for 3 days at 4 °C as previously described^28^. Primary antibodies included: mouse anti-TUJ-1 (1:1000, BioLegend), goat anti-BRN3b (1:250, Santa Cruz), rabbit anti-TH (1:1000 Millipore), rabbit anti-pJUN (1:250, Cell Signaling), rabbit anti-cCASP3 (1:1000, Millipore), and rabbit anti-Neurofilament light chain (1:100; Cell Signaling). Whole retinas were then washed in 1× PBS and incubated in Alexafluor-conjugated secondary antibodies for 2 days at room temperature. Whole retinas were then mounted RGC side up in Fluorogel in TRIS buffer. RGCs were quantified in eight equally spaced 40× fields taken approximately 220 μm from the peripheral edge of the retina as previously described^29^. Quantification was completed using the cell counter tool in ImageJ. The area of fasciculations in *Mkk7*-deficient mice was determined by tracing the outside edge of each fasciculation using the outline tool in Axiovision software (Carl Zeiss).

### Protein extraction and western blotting

Western blots for pJUN in WT and *Mkk4-* and *Mkk7*-deficient mice were completed as previously described^7^. Nanodrop was used to determine protein concentration from retinal lysates and 30 μg of protein was loaded into each well. Blots containing rabbit anti-pJUN (1:500, Cell Signaling) and rabbit anti-GAPDH (1:2000, Cell Signaling) were incubated overnight at 4 °C. A chemiluminescent kit (Supersignal West Dura Extended Substrate, Pierce 34075, Bio-Rad 170–5070) was used to detect immunoreactive bands followed by densitometric analysis using Quantity One Software (Bio-Rad). Western blots for MKK4 in *Mkk4*-deficient mice, MKK7 in *Mkk7-*deficient mice, and pJNK in *Mkk4/Mkk7*-deficient animals were performed as previously described^8^. Briefly, retinas were placed in 100 μl ice-cold lysis buffer after dissection (1× RIPA buffer (Santa Cruz, 24948) and protease/phosphatase inhibitor cocktail (Cell Signaling, 5872S)). Tissue was sonicated (Bransa Digital Sonifier) and spun down in a microcentrifuge. Then, 10 μl of supernatant was boiled for 10 min with 10 μl of 2× Laemmli loading buffer (Bio-Rad) and then run on a 12% sodium dodecyl sulfate–polyacrylamide gel electrophoresis (SDS-PAGE) gel. Membranes were treated with the Qentix Western Blot Signal Enhancer kit (Thermo Scientific, 21050) after they were transferred to a polyvinylidene difluoride (PVDF) membrane and then blocked and probed overnight at 4 °C with primary antibodies: rabbit anti-pJNK (1:500, Cell Signaling), rabbit anti-MKK4 (1:500, Cell Signaling), rabbit anti-MKK7 (1:500, Cell Signaling), or rabbit anti-GAPDH (1:2000, Cell Signaling). Membranes were then washed and treated with secondary antibody: horseradish peroxidase-conjugated anti-rabbit IgG (1:5000, Bio-Rad). A chemiluminescence kit (Immun-star, Bio-Rad 170–5070) was used to detect immunoreactive bands prior to exposure using either film or digital detection equipment (Azure Biosystems c500). Occasionally, membranes were stripped (buffer: 0.1 M Tris-Cl pH 6.8, 2% SDS, 0.7% β-mercaptoethanol) after development and treated with another primary antibody. ImageJ was used to perform densitometric analyses to determine the relative abundance of protein expressed relative to those of glyceraldehyde 3-phosphate dehydrogenase (GAPDH) loading controls.

### Mechanical optic nerve injury

Controlled optic nerve crush (CONC) was performed as previously described^28,30^. Briefly, animals were anesthetized with 100 mg/kg ketamine and 10 mg/kg xylazine, the optic nerve was surgically exposed, and then crushed for 5 s with a pair of self-closing forceps immediately behind the globe. A cohort of eyes underwent sham surgery, in which the optic nerve was exposed but not crushed. These eyes along with eyes that had not been manipulated served as experimental controls. Animals were harvested after 2 h, 1 day, 5 days, or 35 days.

### Statistical analysis

At least three retinas of each genotype were analyzed for all experimental conditions. Experimenters were masked to genotype and experimental condition for all quantification of RGC counts. Unpaired Student’s *t*-tests were used to compare differences across two groups. A one-way analysis of variance followed by the Bonferroni post hoc test for group comparisons was used to compare differences across more than two groups at a single time point. A *P* value < 0.05 was considered statistically significant. Means ± SEM are displayed in graphs.

## Results

### Deficiency of *Mkk4* or *Mkk7* leads to mild alterations in retinal structure

**Fig. 1:**
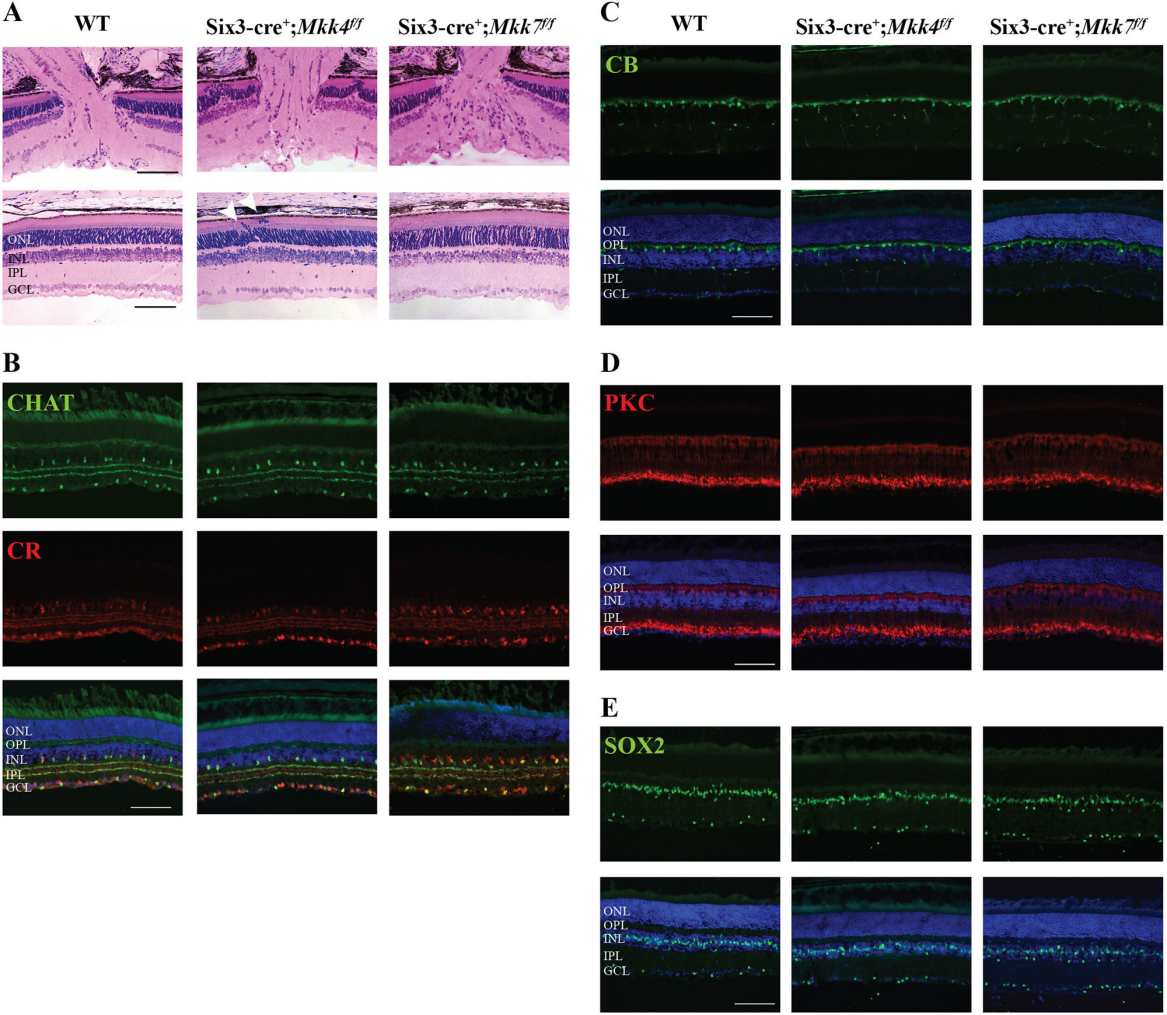
Adult *Mkk4*- and *Mkk7*-deficient retinas exhibit presence of all major cell types with minor structural alterations. **a** Representative 3 μm plastic sections stained with H&E demonstrate areas of photoreceptor cell body attachment to retinal pigmented epithelium in *Mkk4*-deficient retinas (arrowheads). Misplaced cells are also seen in the inner plexiform layer in *Mkk4-*deficient retinas. Retinal lamination in *Mkk7-*deficient retinas appeared grossly normal, though regions of the optic nerve head displayed slightly increased cellularity in the prelaminar region. Antibodies against ChAT and calretinin revealed normal development of cholinergic and AII amacrine cells, respectively, in both *Mkk4-* and *Mkk7-*deficient retinas (**b**). Calbindin-D-28K-labeled horizontal cells (**c**), PKCα-labeled bipolar cells (**d**), and SOX2-labeled Müller glia (**e**) in *Mkk4* and *Mkk7* mutants were furthermore indistinguishable from controls. Merged images with DAPI (blue) are shown below. *N* ≥ 4 retinas for each genotype. Scale bars: 100 μm

To generate retinas deficient in *Mkk4* or *Mkk7*, *Six3*cre was used to recombine floxed alleles of either *Mkk4* or *Mkk7* with efficient deletion of both *Mkk4* and *Mkk7* (>95% and >85% protein reduction, respectively, Fig. S1)^27^. To determine if deletion of *Mkk4* or *Mkk7* is necessary for retinal development, sections of adult WT, *Mkk4-*deficient, and *Mkk7*-deficient retinas were evaluated (Fig. 1a). The *Mkk4-*deficient retina and optic nerve head had grossly normal morphology, apart from occasional photoreceptor cell bodies that were misplaced among photoreceptor inner/outer segments and more cells in the inner plexiform layer. The *Mkk7-*deficient retinas had increased cellularity in the surface nerve fiber layer and prelaminar region of the optic nerve head. Retinal lamination in *Mkk7*-deficient animals appeared normal, although there was slight disruption in the contour of the inner limiting membrane and ganglion cell layer.

To further examine if *Mkk4* and *Mkk7* were necessary for retinal development, immunohistochemistry was used to study specific retinal cell types. Antibodies against choline acetyltransferase (ChAT) and calretinin were used to label amacrine cell bodies in the inner nuclear layer and synaptic strata in the inner plexiform layer^32^. Amacrine cells and inner plexiform lamination in both *Mkk4*- and *Mkk7-*deficient retinas appeared to develop normally (Fig. 1b). Horizontal cells and bipolar cells, stained with calbindin-D-28K and protein kinase Cα (PKCα), respectively, also appeared normal (Fig. 1c, d). Finally, there were no observable differences between WT and *Mkk4-* or *Mkk7*-deficient retinas in SOX2 staining (Müller glia and a subset of amacrine cells, Fig. 1e). Together, these data suggest that aside from sporadic photoreceptor nuclei directly abutting the  retinal pigment epithelium in the outer nuclear layer, all major cell types within the inner nuclear layer appeared to differentiate in *Mkk4*- and *Mkk7*-deficient retinas.

### Adult *Mkk4*- and *Mkk7*-deficient animals have fewer RGCs

**Fig. 2:**
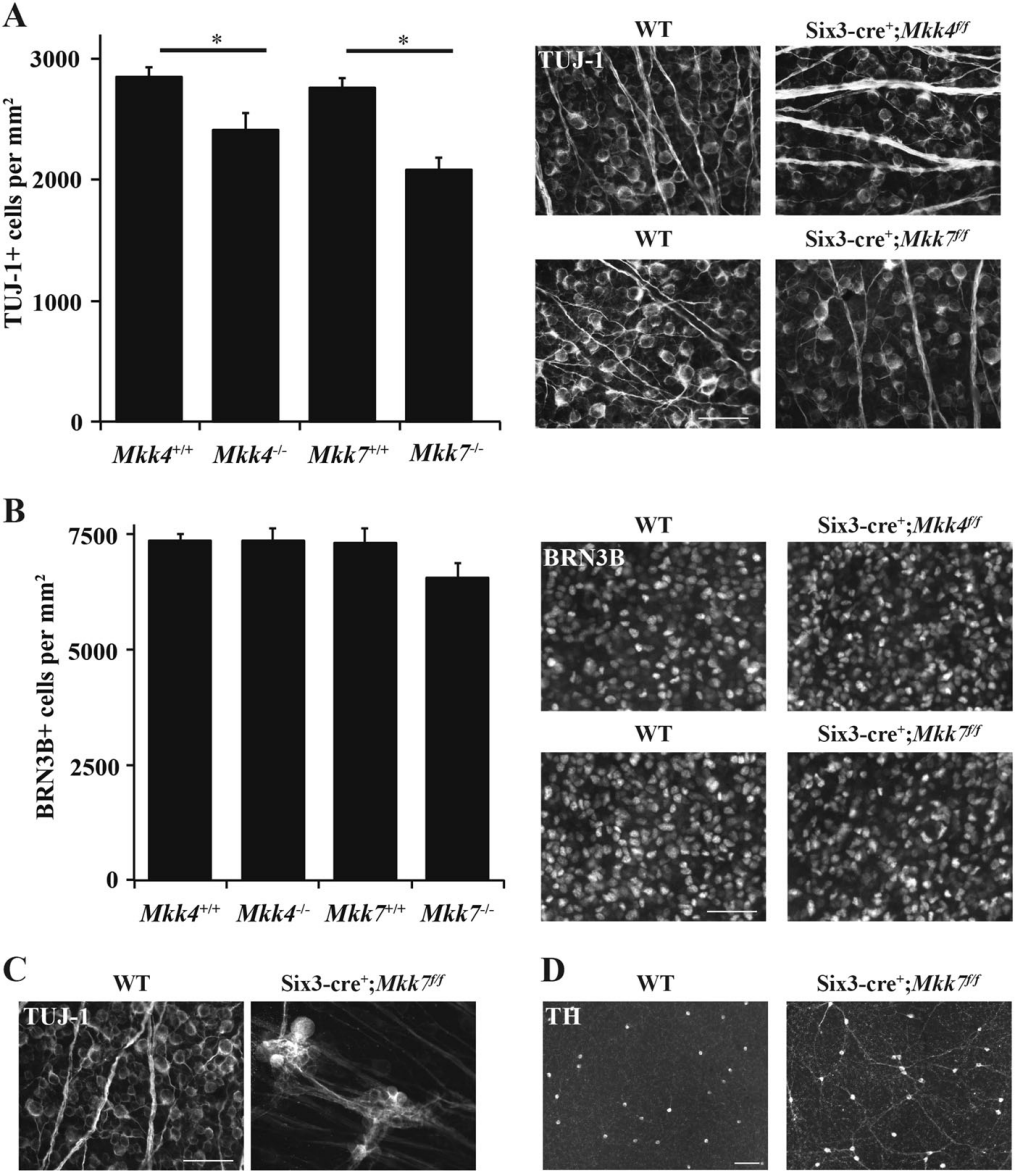
Adult *Mkk4-* and *Mkk7*-deficient animals have fewer retinal ganglion cells resulting from postnatal dropout. **a** The number of TUJ-1-positive RGCs is significantly reduced in adult *Mkk4*- and *Mkk7-*deficient retinal flat mounts. Representative flat mounts are displayed to the right of the graph. (*P ≤ 0.05). *N* ≥ 10 retinas per genotype. **b** P0 flat mounts stained with BRN3B reveal normal RGC densities in both *Mkk4-* and *Mkk7-*deficient retinas, implying RGC dropout occurs postnatally. Representative flat mounts shown to the right of the graph. *N* ≥ 4 retinas per genotype. All examined *Mkk7-*deficient adult retinas displayed areas of abnormal fasciculation in both TUJ-1-positive RGCs (**c**; *N* ≥ 12) and tyrosine hydroxylase (TH)-positive amacrine cells (**d**; *N* ≥ 4). Scale bars: (**a**–**c**) 50 μm; (**d**) 100 μm. Error bars represent SEM

The cellularity of the ganglion cell layer appeared less in the *Mkk4-* and *Mkk7*-deficient retinas than in controls (Fig. 1a). There also appeared to be some disorganization of the nerve fiber layer or inner limiting membrane in the *Mkk7-*deficient retina. Therefore, the number of TUJ-1+ RGCs was counted in retinal flat mounts (Fig. 2a). Retinas contained 15% and 25% fewer RGCs in *Mkk4-* and *Mkk7-*deficient animals, respectively, as compared to controls (*P* < 0.05). Furthermore, while no gross alteration was observed in *Mkk4*-deficient RGCs, *Mkk7* deficiency resulted in sporadic clumping and axonal fasciculation (discussed below; experimenters performing cell counts avoided these small areas). To determine whether the decreased RGC density in adult *Mkk4-* and *Mkk7-*deficient retinas was due to an early developmental defect, flat mounts were examined at P0, a time point subsequent to RGC birth and determination^33^. Retinas were stained for BRN3B (POU4F2), another marker for RCGs^34,35^. RGC cell counts in both mutant mice were normal at this age, suggesting the correct amount of RGCs were born but died at a later stage (Fig. 2b).

### Deficiency of *Mkk7* causes axonal fasciculation in RGCs and dopaminergic amacrine cells

RGC cell body clumping and axonal fasciculation was observed in 100% of *Mkk7-*deficient retinas examined and never observed in *Mkk4*-deficient retinas (Fig. 2c). Areas of affected retina in *Mkk7*-deficient animals comprised only a small portion of the ganglion cell layer (0.91% ± 0.32% of the retina, 0.142 ± 0.05 mm^2^, *n* = 8; Fig. S4A). Aside from the few areas of clumping and fasciculation, which appeared to be randomly distributed throughout the retina, RGC structure in *Mkk7*-deficient retinas appeared similar to WT controls (range: 1–4 areas of fasiculation per retina, mean: 2.5 areas of fasciculation, *n* = 8).

JNK1 is required for Netrin-1-induced axon outgrowth in the spinal cord^36^. Down syndrome cell adhesion molecule (DSCAM) has also been implicated in JNK1–Netrin-1-mediated neurite growth, and its loss of function leads to clustering of both RGC axons and cholinergic amacrine cell dendrites^36,37^. To examine whether the same amacrine cell phenotype resulted from *Mkk4* or *Mkk7* deficiency, additional retinal flat mounts were stained for tyrosine hydroxylase (TH), which labels a subset of dopaminergic amacrine cells. Similar to *Dscam* mutant mice, deficiency of *Mkk7* resulted in fasciculation of dopaminergic amacrine cell dendrites (Fig. 2d) which was far less pronounced within *Mkk4*-deficient retinas (data not shown).

### Deficiency of *Mkk4* or *Mkk7* does not prevent JNK–JUN signaling after axonal injury in RGCs

**Fig. 3:**
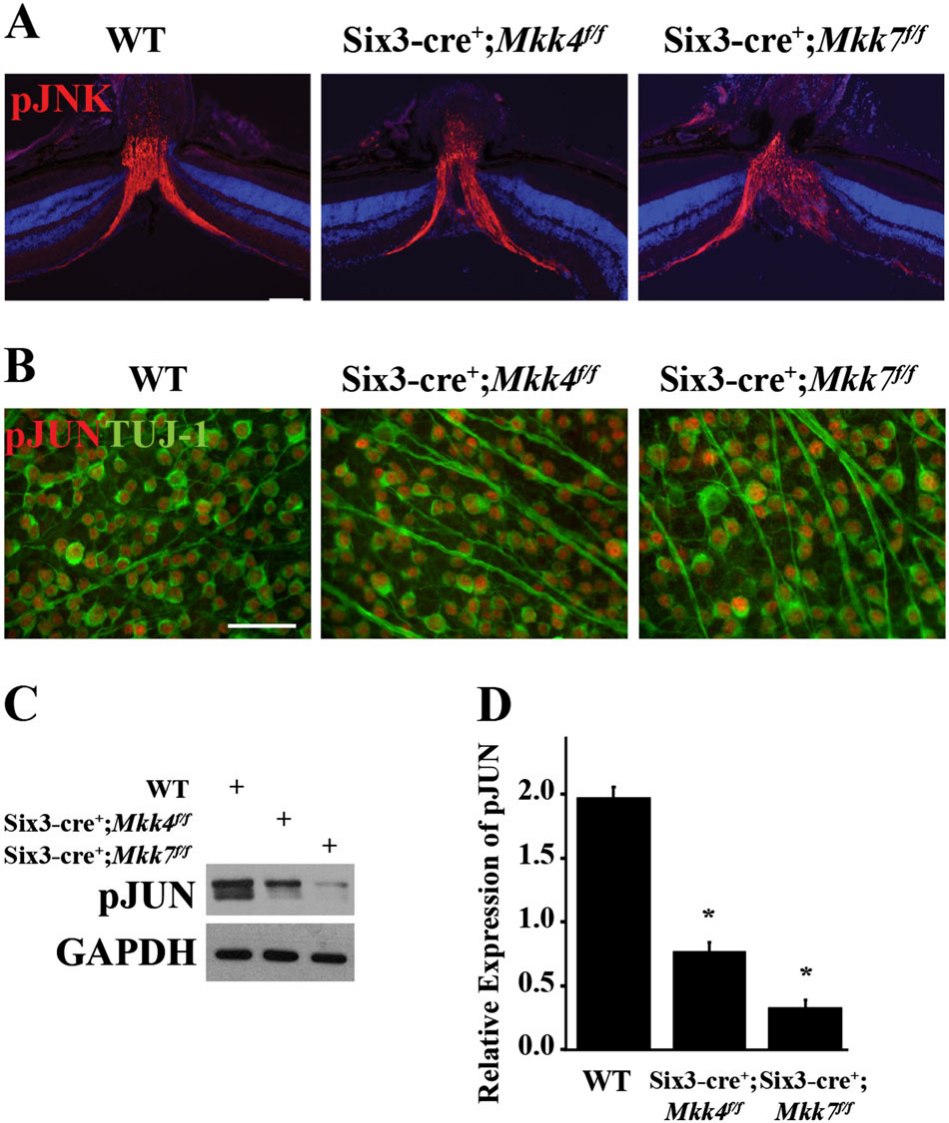
Deficiency of *Mkk4* or *Mkk7* does not prevent JNK–JUN signaling after axonal injury in RGC somas or axons. **a** Representative sections containing the optic nerve head demonstrate robust expression of pJNK 2 h after CONC in WT controls and in *Mkk4* and *Mkk7* mutants (*N* ≥ 3 per genotype). **b** Representative retinal flat mounts from each group reveal robust levels of pJUN in TUJ-1-positive RGCs 1 day after CONC (*N* ≥ 3 per genotype). Scale bars: 50 μm. **c** Representative western blots showing levels of pJUN protein in WT and *Six3*cre driven conditional knockouts after CONC. **d** pJUN protein levels were significantly reduced by 61.8% in *Mkk4*- and by 83.1% in *Mkk7-*deficient mutants compared to littermate WT controls (*N* ≥ 3 per genotype, *P < 0.001)

MKK4 and MKK7 are the only two MAP2Ks that activate JNK, which in turn activates its conical target, JUN. JNK–JUN signaling is important for pro-apoptotic signaling after axonal injury^6,7,38–41^. MKK4 and MKK7 have been previously reported to serve non-redundant functions in vivo^18,26,42–44^. For example, activation of MKK4 but not MKK7 triggers neuronal death following oxidative stress^45^ while MKK7 is required for JNK activation caused by pro-inflammatory cytokines (such as tumor necrosis factor-α and *interleukin*-1)^15^. Together, these results suggest activation of JNK after axonal injury may be preferentially controlled by a single MAP2K. To test this possibility, JNK–JUN signaling was evaluated in the *Mkk4*- and *Mkk7-*deficient animals. Both JNK and JUN are activated following optic nerve injury^7,10,46,47^. Expression of pJNK was observed in RGC axons entering the optic nerve head and pJUN-positive RGCs were present in WT and *Mkk4*- and *Mkk7*-deficient mice (Fig. 3a, b). Levels of pJUN were significantly reduced in both *Mkk4-* and *Mkk7* (*P* < 0.001, *N* ≥ 3 for each cohort)-deficient retinas after CONC; however, detectable levels of the protein were found in both cohorts (Fig. 3c, d). No pJUN was detected in the sham condition in any animal cohort (data not shown). Thus, neither *Mkk4* nor *Mkk7* are independently required for activation of JNK or JUN after axonal injury.

### Deficiency of *Mkk4* or *Mkk7* protects RGCs after acute mechanical axonal injury

**Fig. 4:**
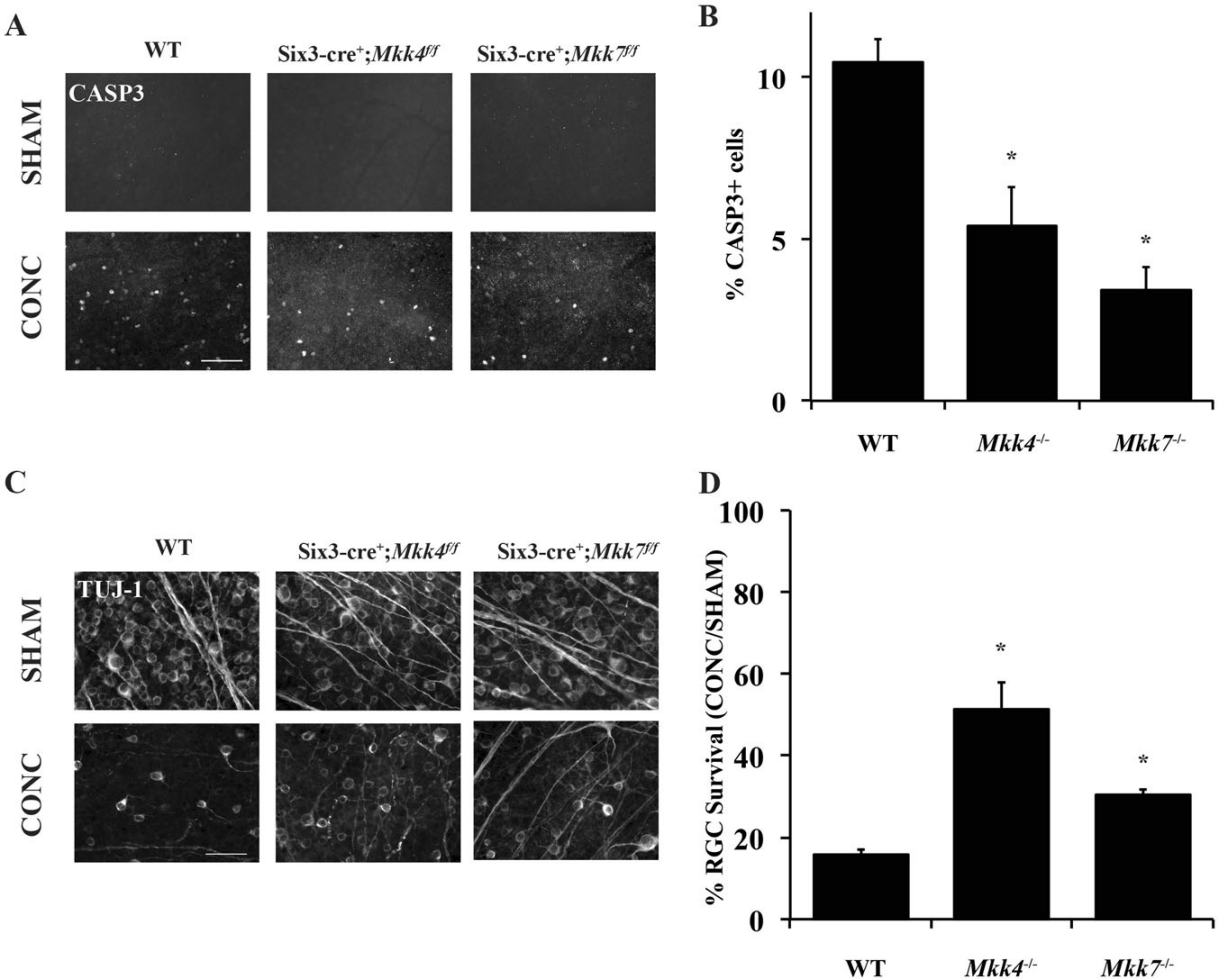
Deficiency of *Mkk4* or *Mkk7* provides moderate RGC protection after CONC. **a** Representative retinal flat mounts stained with cleaved caspase-3 (CASP3) in eyes 5 days after CONC or sham surgery. **b** Significantly fewer apoptotic cells are present in *Mkk4-* and *Mkk7-*deficient animals as compared to WT controls following CONC. *N* ≥ 4 per genotype per condition; *P ≤ 0.05. **c** Representative retinal flat mounts stained with TUJ-1 following CONC or sham surgery. **d** Significantly more cells remain 35 days after CONC in *Mkk4-* and *Mkk7*-deficient retinas as compared to WT controls. *N* ≥ 8 per genotype per genotype and condition. Scale bars: 50 μm. Error bars represent SEM

The MAPK signaling family has been previously shown to be an important pro-apoptotic signaling pathway after axon injury ^6,7,38–41^. To determine if *Mkk4* and/or *Mkk7* is critical for axonal injury-mediated RGC death, RGC survival was analyzed in mice deficient in *Mkk4* and *Mkk7* after CONC. *Mkk4-* and *Mkk7-*deficient retinas had significantly fewer dying RGCs (cleaved caspase-3-positive cells) at 5 days after CONC as compared to controls even after correcting for the decreased number of RGCs in *Mkk4-* and *Mkk7-*deficient retinas (Fig. 4a, b). In addition, at 35 days after CONC, *Mkk4*- and *Mkk7-*deficient retinas had a small but significantly greater percentage of surviving RGCs as compared to controls (Fig. 4c, d; % survival ± SEM: WT, 16.0% ± 1%; *Mkk4*, 51.5% ± 6.4%; *Mkk7*, 30.4% ± 1.4%). This protection was not as robust as that previously observed in *Jun-*deficient mice (~75%) at the same time point, suggesting both *Mkk4* and *Mkk7* can activate JNK–JUN-dependent RGC death after axonal injury.

### Combined deficiency of *Mkk4* and *Mkk7* leads to severe alterations in retinal structure

**Fig. 5:**
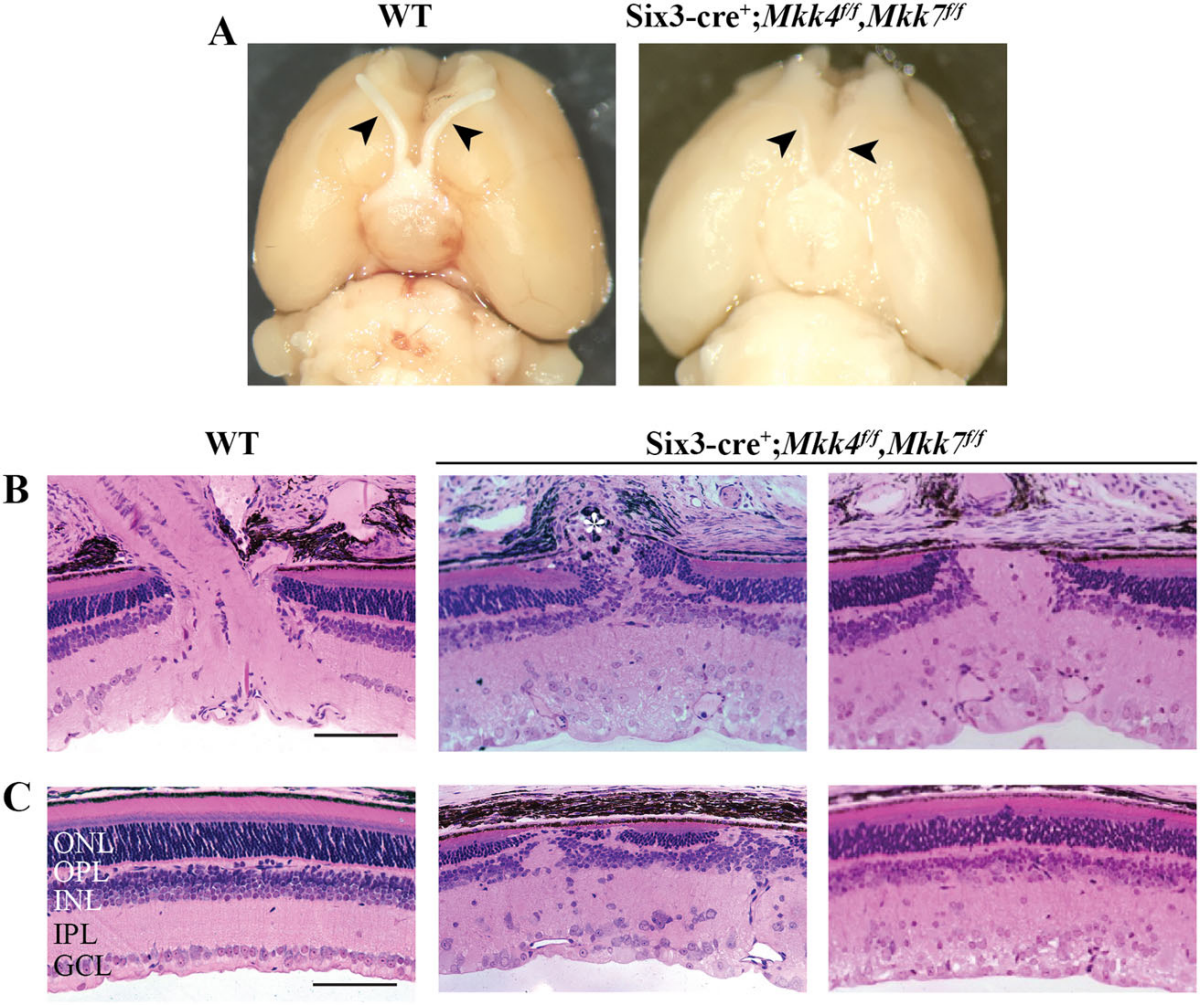
Combined deficiency of *Mkk4* and *Mkk7* causes failure of optic nerve formation and disruption of retinal lamination. **a** Brains were removed from adult WT and *Mkk4/Mkk7* double-deficient mice with optic nerves intact (arrowheads). Near complete loss of both optic nerves was evident in all *Mkk4/Mkk7* mutant brains examined (*N* = 8 per genotype). **b** Representative 3 μm plastic sections stained with H&E further demonstrate severe optic nerve head abnormalities (asterisk) in *Mkk4/Mkk7* double-deficient mice eyes (*N* = 4 per genotype). **c** *Mkk4/Mkk7* double mutants display extensive disruption of all retinal layers (*N* = 4 per genotype). Scale bars: 100 μm

**Fig. 6:**
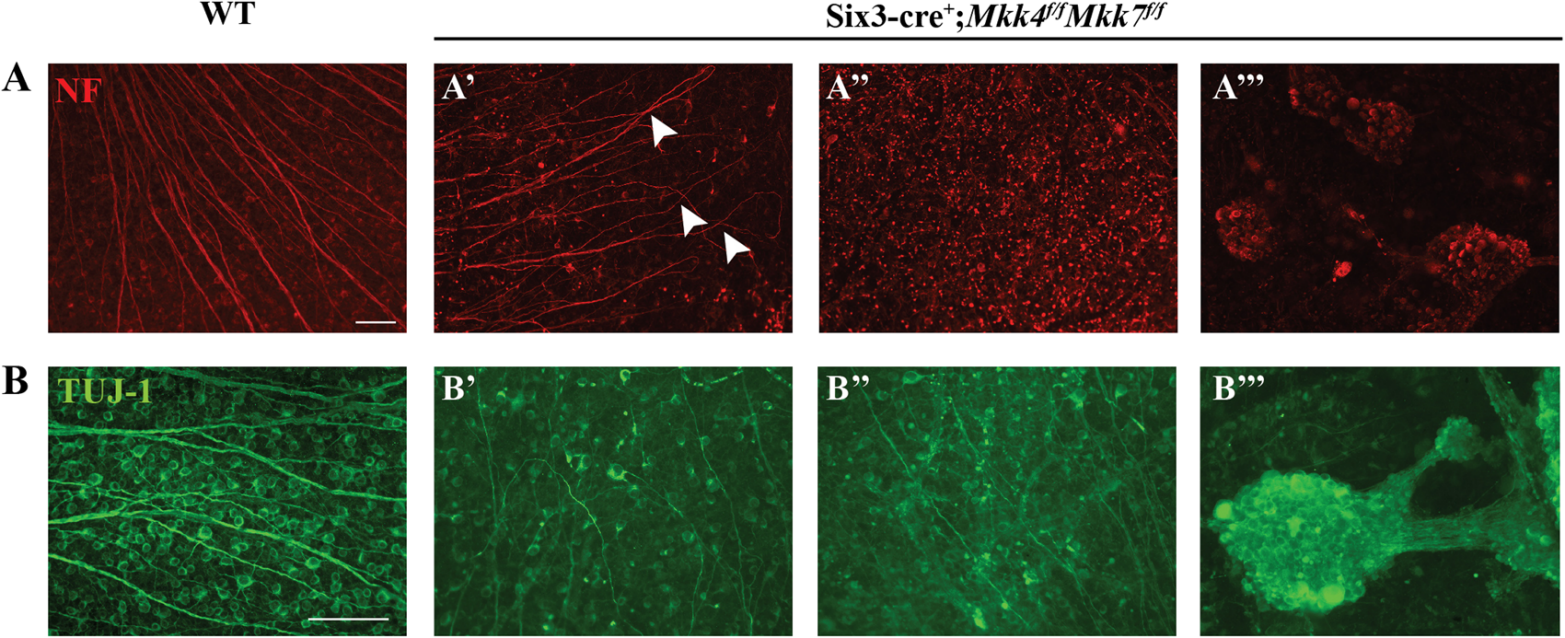
Fasciculation of RGCs and amacrine cells in retinas of *Mkk4/Mkk7* double-deficient mice. Retinal flat mounts were stained with neurofilament (NF; **a**) and TUJ-1 (**b**) to assess RGC organization. *Mkk4/Mkk7* double-deficient mutants displayed phenotypic variability in RGC structure, in which some areas exhibited axonal overlap (arrowheads in **a’**) and irregular axonal trajectories (**b’**), other areas appeared relatively normal (**a”**), and several areas consisted of RGC clumps (**a”’**, **b”’**). *N* ≥ 4 per genotype. Scale bars: 100 μm

Deficiency of *Mkk4* or *Mkk7* alone provided significant protection to RGCs after mechanical axon injury; however, neither deficiency by itself afforded complete protection to RGCs. As MKK4 and MKK7 are the only known molecules acting directly upstream of JNK, MKK4 and MKK7 appear to have redundant roles in RGCs after CONC^18,43^. Moreover, as either deficiency alone did not cause severe retinal dysgenesis during development, removing both genes, and therefore any possible genetic redundancy, could allow for the role of MAP2Ks in axonal injury-induced RGC death to be tested. To examine these possibilities, retinas deficient in both *Mkk4* and *Mkk7* were generated using *Six3*cre. JNK signaling, as measured by pJNK protein levels, was reduced by 86.7% in dual *Mkk4/Mkk7*-deficient mice (Fig. S3). The remaining pJNK in the *Mkk4/7* dual-deficient animals is likely the result of incomplete recombination by *Six3*cre (known to occur in approximately 20% of RGCs)^8^. *Mkk4/Mkk7*-deficient mice had highly atrophic optic nerves (Fig. 5a), disrupted optic nerve head morphology (Fig. 5b), and disorganized ganglion cell layer pathology (Fig. 5c). The lack of an optic nerve prevented analysis of axonal injury-induced death in *Mkk4/Mkk7*-deficient mice. Surprisingly, despite the lack of an optic nerve, *Mkk4/Mkk7-*deficient retinas still had TUJ-1+ RGCs. However, there was clear evidence of abnormalities in RGC organization. Some areas of *Mkk4/Mkk7*-deficient retinas displayed abnormal RGC axonal crossing, while other areas appeared relatively normal (Fig. 6a). The *Mkk4/7*-deficient animals also displayed an RGC clumping phenotype (both in the periphery and near the optic nerve head) which was more prevalent and severe than the phenotype observed in *Mkk7-*deficient retinas (Fig. 6b and Fig. S4). Due to the widespread abnormalities in the *Mkk4/Mkk7*-deficient retinas, direct quantification of total RGC counts or the specific area of RGC fasciculation was not feasible.

**Fig. 7:**
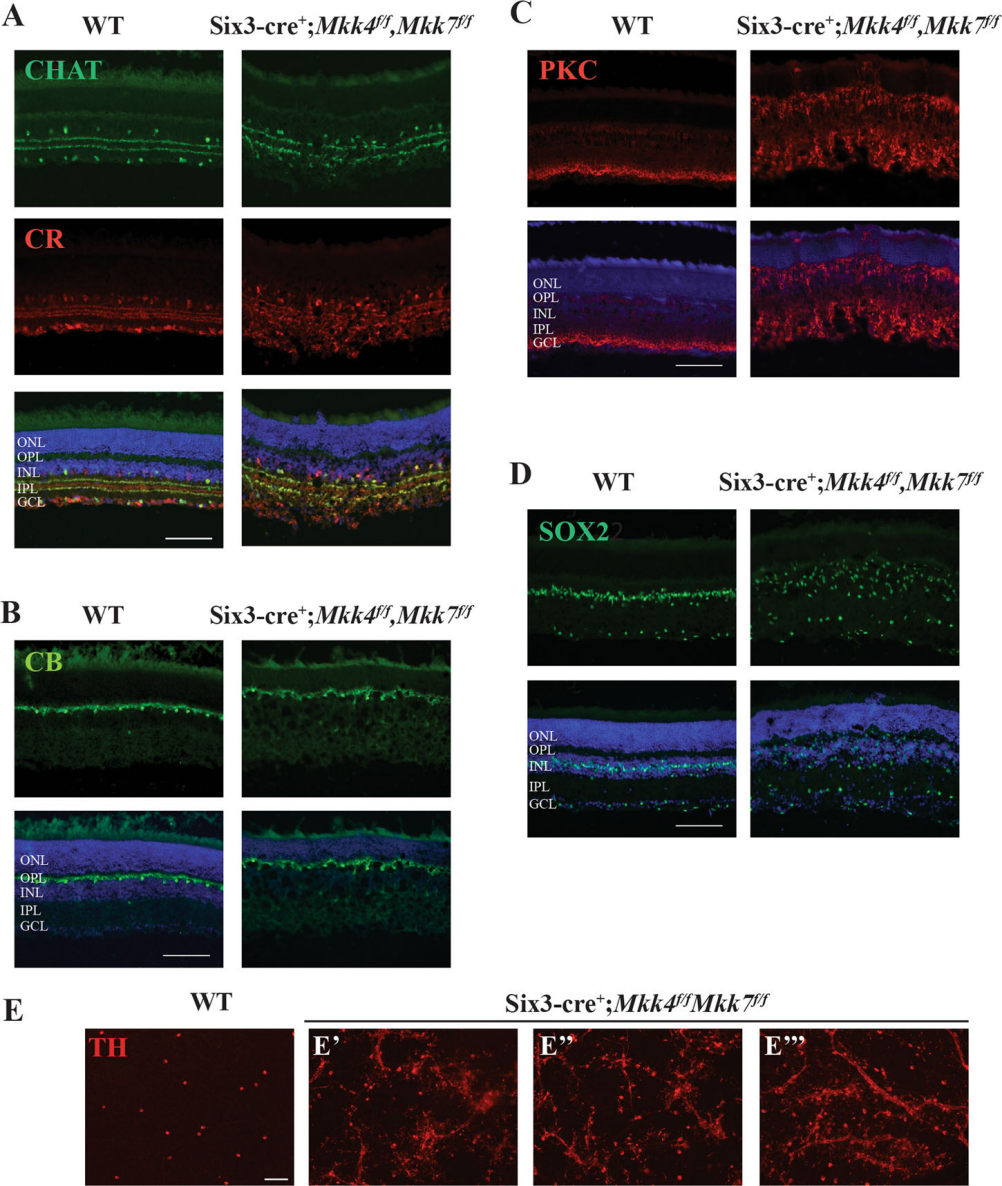
Combined deficiency of *Mkk4* and *Mkk7* leads to severe alterations in retinal structure. Representative sections of WT and *Mkk4/Mkk7-*deficient retinas stained with specific cell markers show clear disorganization of amacrine cells (**a**), slight defects in horizontal cells (**b**), and abnormal distribution and morphology of bipolar cells (**c**) and Müller glia (**d**) in retinas of *Mkk4/Mkk7* double-deficient mutants. Merged images with DAPI (blue) are shown below. *N* ≥ 4 per genotype. **e** *Mkk4/Mkk7* double-deficient flat mounts further display clumping and dendritic fasciculation of dopaminergic amacrine cells. *N* ≥ 4 per genotype. Scale bars: 100 μm

Retinal sections also showed severe abnormalities in retinal lamination (Fig. 5c). These abnormalities included areas of retinal photoreceptor thinning and areas of hyper- and hypoplasia in the inner nuclear layer. Overall, each layer of the retina appeared thinner compared to WT controls, and multiple cell types were misplaced. Interestingly, histological retinal phenotypes did not appear to worsen with age, as 9-month-old *Mkk4/Mkk7*-deficient mice had similar histological irregularities compared to the younger mice analyzed (Fig. S2). To further investigate the nature of the histological abnormalities, immunohistochemistry was used to identify retinal cell types and overall retinal organization. There were clear abnormalities in CHAT+ and calretinin+ amacrine cells. Particularly, these markers showed that the inner plexiform layer had areas of synaptic disruption (Fig. 7a). Horizontal cells labeled with calbindin-D-28K had a mild disruption of somal and dendritic organization (Fig. 7b). PKCα+ bipolar cells had abnormal somal organization in the inner nuclear layer, occasional bipolar cell nuclei present in the photoreceptor layer, and abnormal bipolar cell termination in the inner plexiform layer (Fig. 7c). SOX2+ Müller glia and amacrine cells were highly irregular in their somal distribution (Fig. 7d). Dopaminergic amacrine cell clumping and dendrite fasciculation was more severe in *Mkk4/Mkk7*-deficient retinas than in retinas deficient in only *Mkk4* or *Mkk7* alone as larger areas of clumping occupied all sectors of the retina in the dual *Mkk4/7*-deficient animals (Fig. 7e and Fig. S4D). These data suggest the involvement of compensatory mechanisms by which *Mkk4* and *Mkk7* might serve redundant roles in retinal neurodevelopment. Overall, there was clear disruption of inner nuclear layer cell somal and synaptic organization.

## Discussion

MAPK signaling is important for both retinal development and RGC apoptotic signaling after axonal injury. Herein, we demonstrate MKK4 and MKK7 provide both overlapping and divergent roles in retinal development and injury signaling. Single deficiency of *Mkk4* or *Mkk7* resulted in minor alterations of retinal lamination. RGC counts were significantly lower in adult *Mkk4-* and *Mkk7-*deficient retinas as compared to controls. After axonal injury, JNK signaling persisted in *Mkk4-* and *Mkk7-*deficient retinas; however, pJUN levels were reduced and RGCs were mildly protected from apoptotic cell death in both mutants. To assess redundancy of MKK4 and MKK7 in development and after injury, animals deficient in both *Mkk4* and *Mkk7* were developed. Together, MKK4/MKK7 were found to play critical roles in the development and organization of all major retinal cell types. The severity of optic nerve dysgenesis combined with significant alterations in retinal organization in the *Mkk4/Mkk7*-deficient retinas precluded examination of the contribution of both MKK4/MKK7 to pro-death signaling after axonal injury. Together, these data suggest novel roles for both MKK4 and MKK7 in retinal development and in axonal injury signaling.

### Single *Mkk4* or *Mkk7* deficiency leads to multiple developmental abnormalities in the retina

Deficiency of either *Mkk4* or *Mkk7* led to significantly decreased RGC number in adult retinas, which was not observed at P0. Throughout retinal development, an excessive number of cells are born that must undergo apoptotic pruning. Developmental RGC pruning occurs from P0 to P7, with a second, smaller wave of cell death occurring around P15^48–50^. Given the postnatal dropout of RGCs in *Mkk4-* and *Mkk7*-deficient animals, MKK4 and MKK7 likely contribute to RGC survival during the normal window of programmed RGC death. Previously, the downstream effector molecules of MKK4 and MKK7, the JNKs, have been shown to regulate photoreceptor apoptosis during the final wave of cell death^14^. Thus, it is possible that MKK4 and MKK7 might play a similar role facilitating pruning of RGCs. Deficiencies in axonal transport or insufficient trophic support within the RGCs themselves may result in excessive loss of RGCs in the mature retina of *Mkk4-* and *Mkk7*-deficient mice^48,51^. Mild disruption of cell adhesion molecules may lead to the axonal fasciculations observed in the *Mkk7-*deficient retinas. As axonal fasciculations were not observed in the *Mkk4*-deficient retinas and *Mkk7-*deficient retinas had significantly fewer RGCs, it is plausible that MKK7 plays a more significant role in axonal guidance and retinal pruning. Determining the overlapping and unique downstream effectors of MKK4 and MKK7 that contribute to retinal development will help define the required molecular cues for proper retinal lamination.

### Deficiency in both *Mkk4* and *Mkk7* causes severe retinal dysgenesis

In contrast to the *Mkk4* and *Mkk7* single-deficient retinas, the *Mkk4/Mkk7* dual-deficient retinas had severe alterations in retinal lamination and axonal projections, as well as optic nerve hypoplasia. These data suggest MKK4 and MKK7 have redundant roles in retinal development and that together these molecules play critical roles in retinogenesis. Previously, JNK signaling has been shown to initiate bone morphogenetic protein-4 (BMP4) and sonic hedgehog (SHH)-mediated control of paired-like homeobox transcription factor (PAX2), which contributes to closure of the optic fissure^12,52^. Netrin-1 also contributes to optic nerve head formation as deficiency of *Netrin-1* leads to failure of RGC axons to exit the eye leading to optic nerve hypoplasia^53^. Netrins serve as important axonal guidance cues in the nervous system and contribute to proper axon outgrowth and pathfinding^54^. JNK1 has been shown to be required for netrin-1 signaling and inhibition of JNK1 reduces netrin-1-dependent axonal projections and pathfinding in other neural systems^36^. DSCAM is a netrin-1 receptor necessary for neurite arborization and prevention of abnormal neural fasciculations^55,56^. Axonal fasciculations and ectopic photoreceptor phenotypes similar to those in *Mkk4/Mkk7*-deficient retinas were observed in *Dscam*-deficient retinas^57^. Proper fasciculation is necessary for RGC axon pathfinding; however, as axons approach deeper targets in the brain, they must disassociate from their neighbors^58^. It is possible in *Mkk4/Mkk7* mutants, the extrinsic cues directing axon divergence are disrupted, resulting in abnormal fasciculation in the retina. *Jnk2/3*-deficient mice and *Jun-*deficient mice do not have aberrant lamination or optic nerve head dysgenesis, which further supports the idea that morphologic differences observed in the *Mkk4/Mkk7*-deficient mice are likely due to JNK1 or JUN-independent signaling mechanisms^7,59^. Future study to evaluate the downstream signaling mechanisms of MKK4 and MKK7 will likely reveal key contributors to retinal and optic nerve development. Despite the lack of optic nerve formation in the *Mkk4/Mkk7-*deficient animals, RGCs survive within the retina. Normal pathfinding and neurotropic support is essential for RGC survival, and thus RGC survival in the absence of optic nerve formation may be due to decreased pro-apoptotic signaling as JNK–JUN signaling contributes to both pro-survival and pro-apoptotic signaling^14,21,60–65^.

### MKK4 and MKK7 are both involved in axonal injury-induced RGC death

Pro-apoptotic MAPK signaling is also an important component of molecular signaling after axonal injury in RGCs^6–8,21–24,39–41^. Similar to other MAPK family members, single deficiency of *Mkk4* or *Mkk7* provides significant protection to RGCs after axonal injury. The level of protection observed, however, does not phenocopy deficiency of other MAPK signaling molecules, supported by the significant, but not complete, reduction of pJUN. Deficiency of the upstream MAP3K, *Dlk*, and downstream targets, *Jnk2/3* and *Jun*, provided greater protection to RGCs after CONC than that observed in this study, suggesting MKK4 and MKK7 likely have redundant roles in pro-apoptotic signaling after axonal injury^6,7,39,40,59,66^.

While both MKK4 and MKK7 are known to activate the JNKs, MKK4 additionally regulates p38^4,67^. Activation of p38 occurs after axonal injury in both neurons and glia^11,47,68–70^. Inhibition of p38 signaling has been shown to provide mild protection to RGC somas and reduce axonal transport deficits following axonal injury^11,68,69^. Activation of the p38 arm of MAPK signaling can also directly activate *Ddit3* (DNA damage inducible transcript 3) which encodes the protein CCAAT/enhancer binding homologous protein (CHOP)/GADD153, a key mediator of endoplasmic reticulum (ER) stress^71,72^. CHOP has been independently shown to be important for pro-apoptotic signaling after axonal injury^59,73,74^. In order to understand the molecular signaling cascade contributing to RGC death after axonal injury, it will be necessary to parse apart the downstream signaling contributions of MKK4 and MKK7.

Future studies should also evaluate combined *Mkk4/Mkk7* deficiency in a temporally controlled conditional knockout animal (allowing for normal retinal and optic nerve development) to determine if deficiency of these molecules together might provide greater protection to RGCs after injury than either alone. It is tempting to consider that dual deficiency of *Mkk4/Mkk7* may be more protective of RGCs than deficiency of other downstream targets alone, as combined deficiency of *Mkk4/Mkk7* may alter multiple molecular signaling pathways that have been previously shown to be important for RGC death including JNK, p38, and ER stress signaling^7,11,59,75^.

Future investigation of the unique and overlapping roles of MKK4 and MKK7 must consider the downstream targets of their activation. MKK4 and MKK7 are known to preferentially target different amino acid residues on the JNKs. MKK4 targets the tyrosine residue while MKK7 preferentially phosphorylates the threonine residue^76,77^. Analysis of the transcriptome of MKK4 and MKK7 is necessary to identify specific downstream targets. Such inquiry would determine if the downstream effectors leading to developmental phenotypes in deficient animals are independent from those downstream effectors mediating pro-apoptotic signaling in RGCs after optic nerve crush.

## Conclusion

MKK4 and MKK7 are members of the MAPK pathway important for both retinal development and the injury response following axonal insult. While we have shown MKK4 and MKK7 are required for maintaining RGC survival, future studies should examine the cellular and molecular mechanisms underlying the observed postnatal dropout in single-deficient animals. Single deficiency of *Mkk4* and *Mkk7* offer mild protection for RGCs against optic nerve injury, though there was likely genetic compensation allowing for pro-apoptotic signaling to proceed. Due to the severity of developmental defects in the double-deficient mice, possible additive protection following mechanical insult was not assessed. Additional experiments employing an inducible double *Mkk4/Mkk7* knockout would allow for proper optic nerve formation and subsequent analysis of RGC survival following injury. This would define the aggregate role of these MAP2K molecules and the specificity of the cellular response in a glaucomatous-relevant injury model.

## Electronic supplementary material

Supplemental Material
