# Author Correction: *Mkk4* and *Mkk7* are important for retinal development and axonal injury-induced retinal ganglion cell death

**DOI:** 10.1038/s41419-018-1233-2

**Published:** 2019-01-25

**Authors:** Stephanie B. Syc-Mazurek, Rebecca L. Rausch, Kimberly A. Fernandes, Michael P. Wilson, Richard T. Libby

**Affiliations:** 1 Department of Ophthalmology, University of Rochester Medical Center, Rochester, NY USA; 2 Neuroscience Graduate Program, University of Rochester Medical Center, Rochester, NY USA; 3 Department of Biomedical Genetics, University of Rochester Medical Center, Rochester, NY USA; 4 The Center for Visual Sciences, University of Rochester, Rochester, NY USA

Correction to: Cell Death and Disease (2018) 9:1095 10.1038/s41419-018-1079-7

There was an error introduced into Figures 4, 5, and 7 during the proofing stage which has since been corrected.

**Fig. 4:**
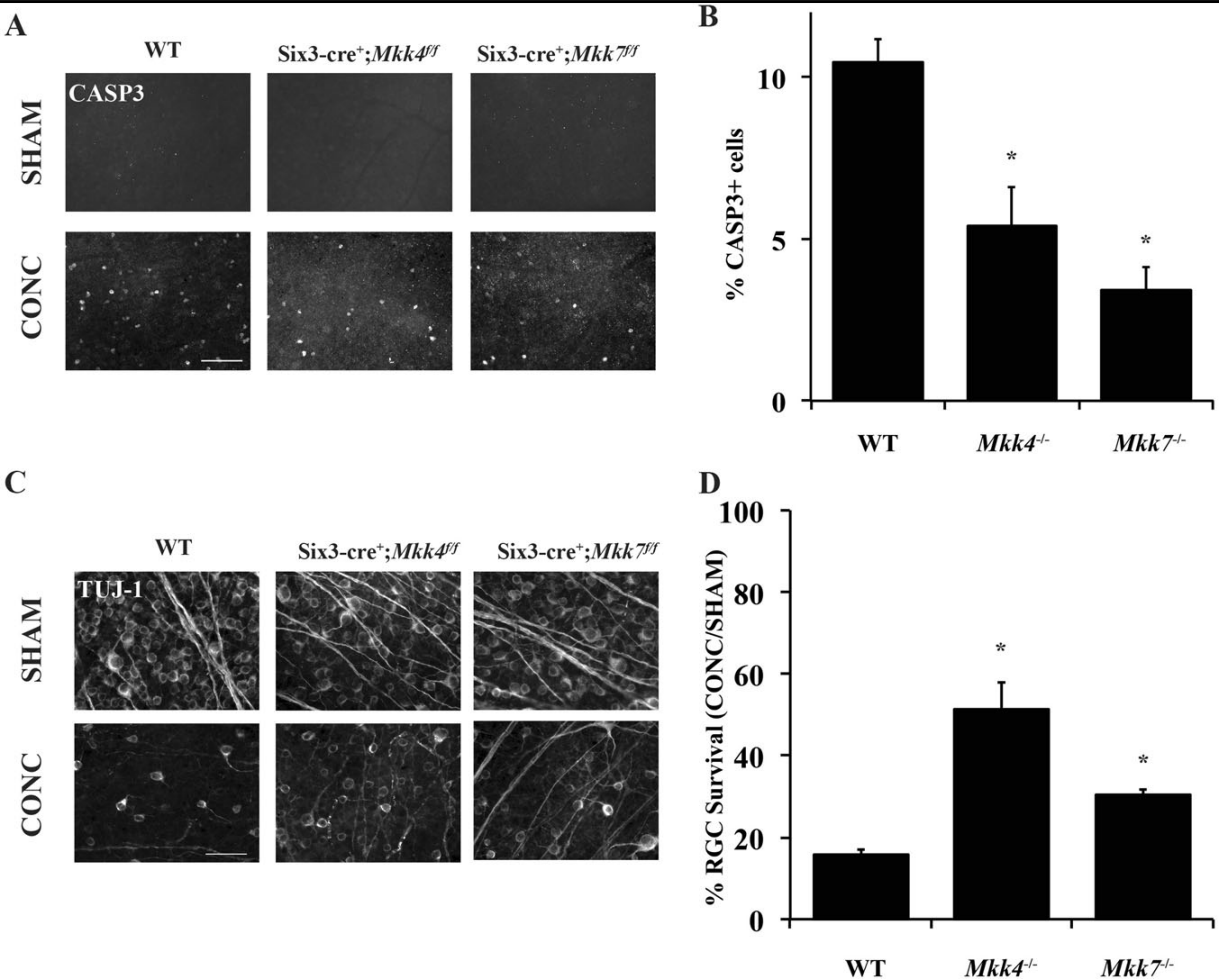
▓

**Fig. 5:**
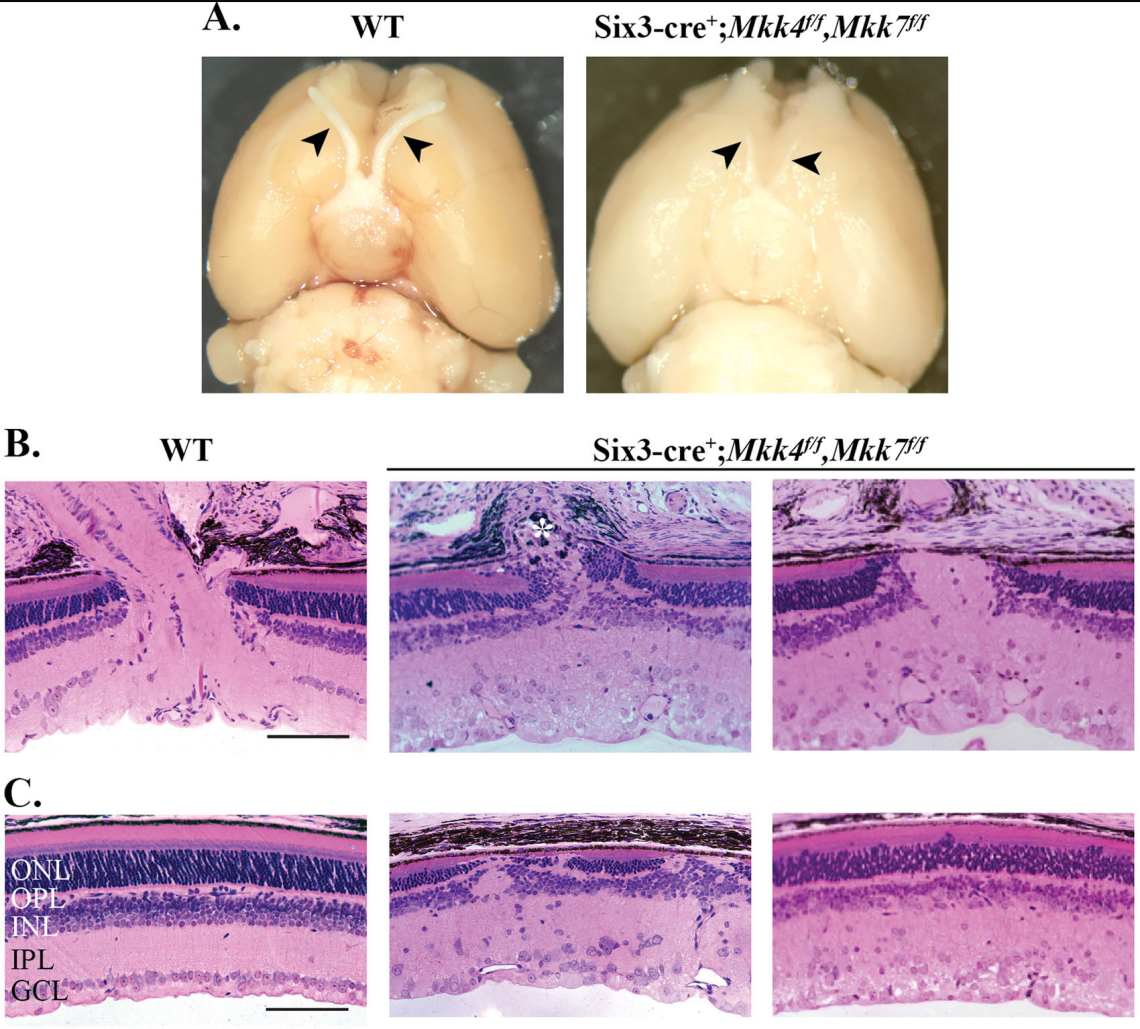
▓

**Fig. 7:**
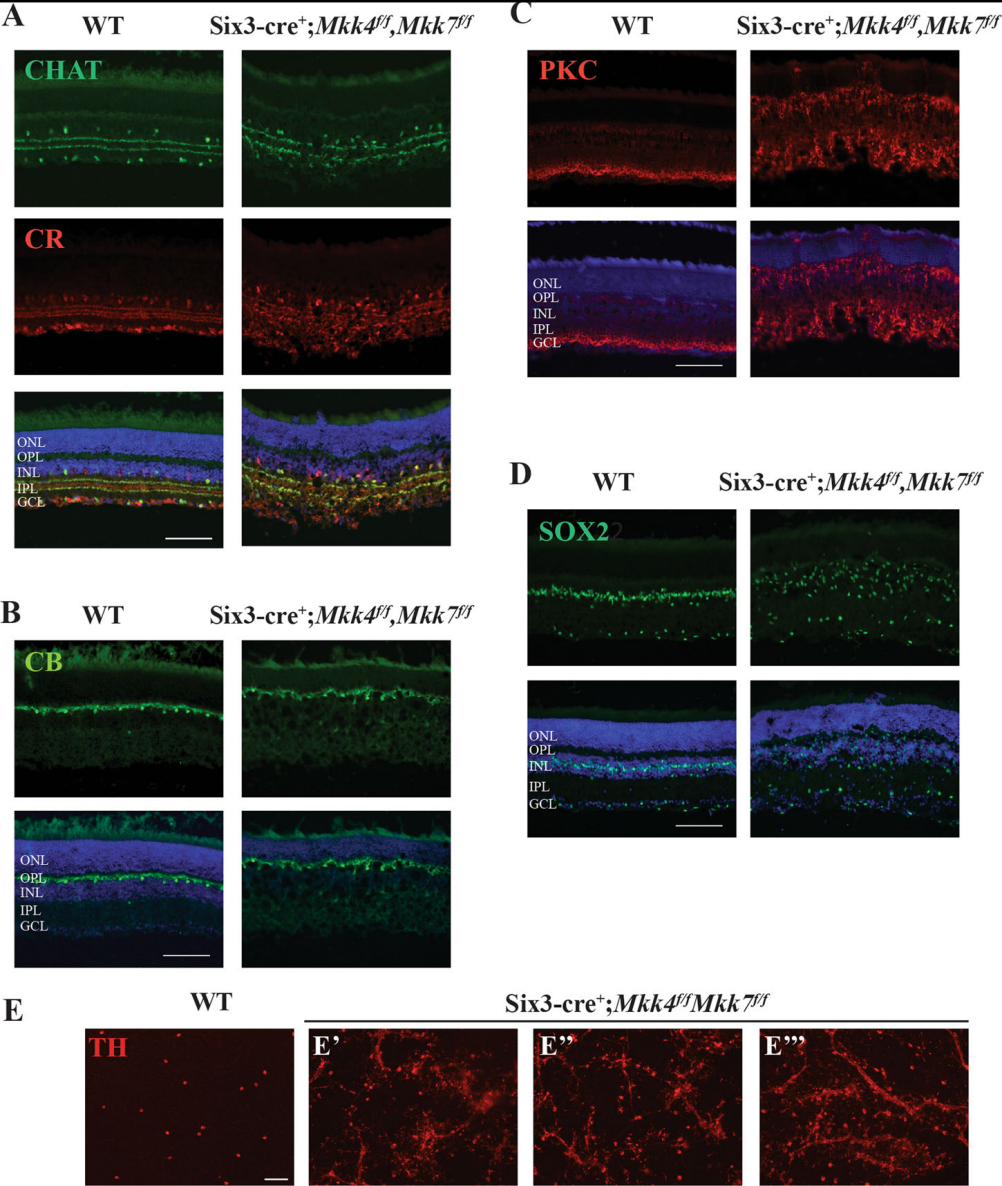
▓

The PDF and HTML versions of the paper have been modified accordingly.

