# Supplementary material for: Mkk4 and Mkk7 are important for retinal development and axonal injury-induced retinal ganglion cell death
Source: Cell Death Dis. 2018 Oct 26;9(11):1095. doi: 10.1038/s41419-018-1079-7 (PMC6203745; doi:10.1038/s41419-018-1079-7)

Supplementary Information

Supplementary Figure S1. Validation of *Mkk4* and *Mkk7* deletion using *Six3*cre.

Representative western blots showing levels of MKK4 and MKK7 protein in respective *Six3*cre driven conditional knockouts. There was an absence of MKK4 protein and a clear reduction of MKK7 protein compared to littermate WT controls (N≥4 per genotype).

Supplementary Figure S2. Retinal lamination in aged *Mkk4/Mkk7* double mutants.

Representative 3µm plastic sections stained with H&E reveal additional retinal deterioration does not occur over time in *Mkk4/Mkk7* deficient mutants aged to 9 months (N=4 per genotype). Extensive disruption of all retinal layers is similar to that observed in *Mkk4/Mkk7* deficient mutants examined at 2 months of age (refer to Fig. 5).

Supplementary Figure S3. pJNK levels in *Mkk4/Mkk7* double mutants.

(A) Representative western blots showing level of pJNK protein in *Six3*cre driven conditional knockouts. **(B)** pJNK protein levels were significantly reduced by 86.7% in *Mkk4/Mkk7* dual deficient mutants compared to littermate WT controls (N≥4 per genotype, p=0.039).

Supplementary Figure S4. Axonal fasciculation and clumping is more prevalent in *Mkk4/Mkk7* double deficient animals than in *Mkk7* deficient animals.

Representative flat mount images of TUJ-1 stained RGCs and RGC axons in *Mkk7* **(A)** and *Mkk4/Mkk7* **(B)** deficient animals. Additional images of neurofilament **(C)** at low and high power and tyrosine hydrolase **(D)** demonstrate widespread abnormalities in axons and dopaminergic amacrine cells in *Mkk4/Mkk7* dual deficient animals respectively. Abnormalities for the

Mkk4/Mkk7 dual deficient animals are observed at the optic nerve head (ONH) and in the peripheral retina. For additional images and wildtype controls please see Figure 6. Scale bars for all images: 100 μm.

Supplementary Figure 1

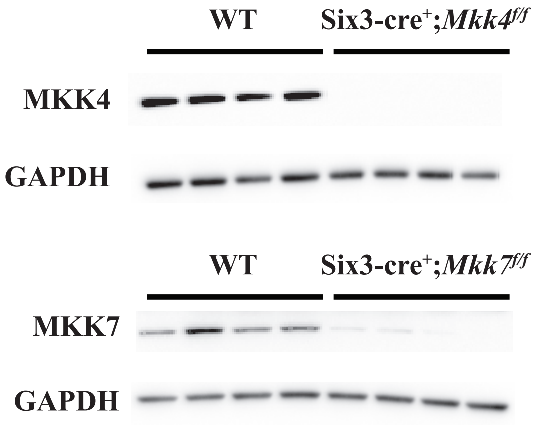

Supplementary Figure 2

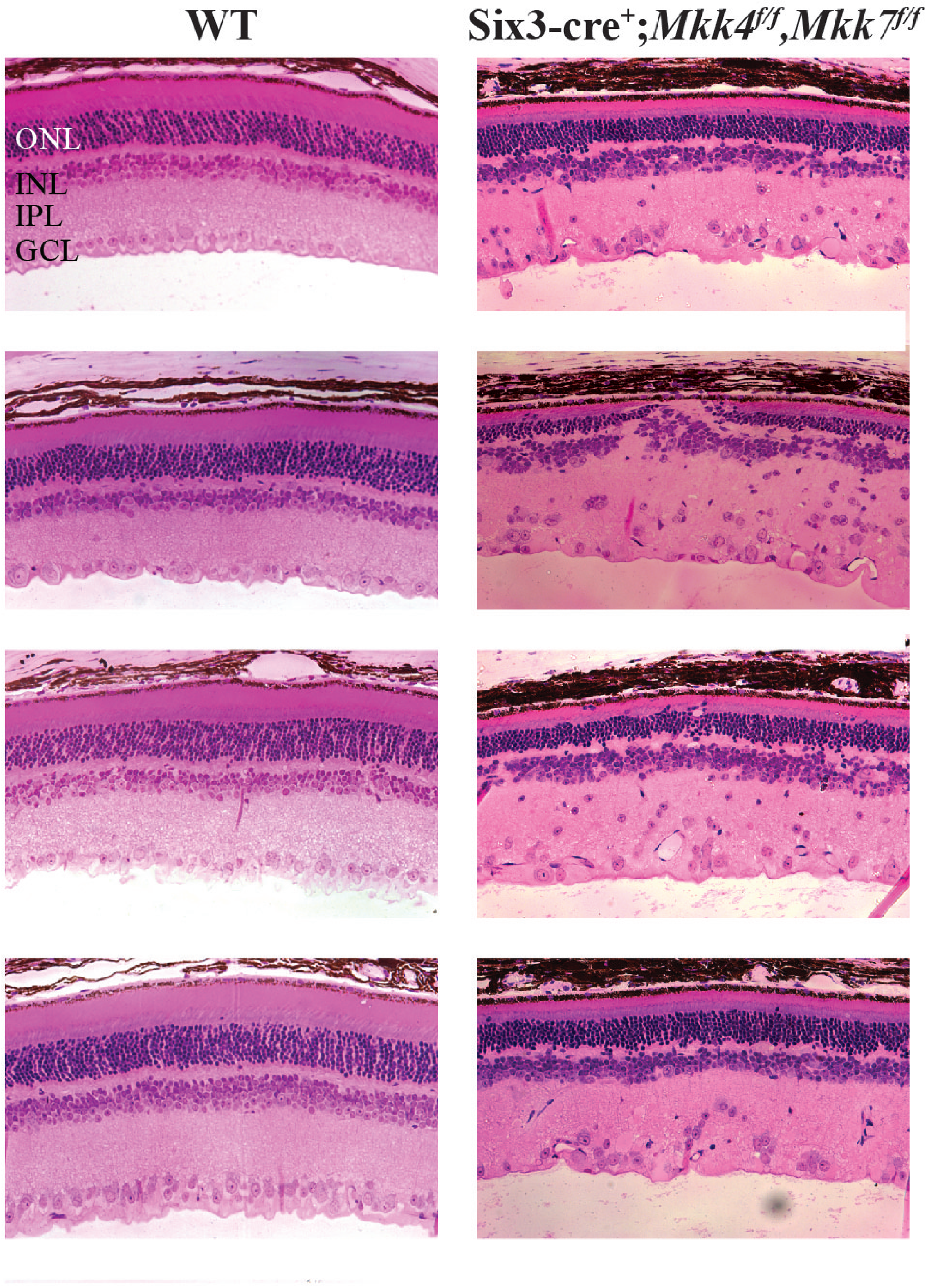

Supplementary Figure 3

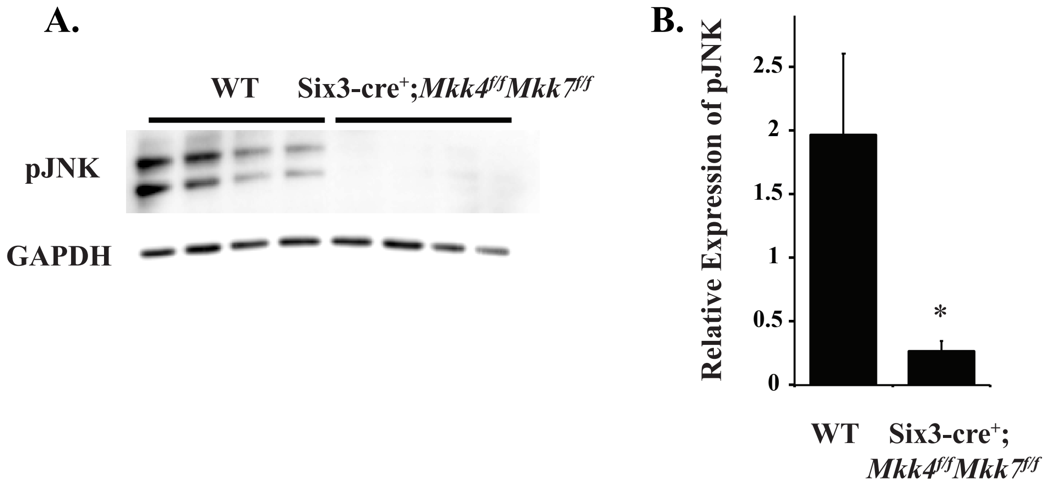

Supplementary Figure 4

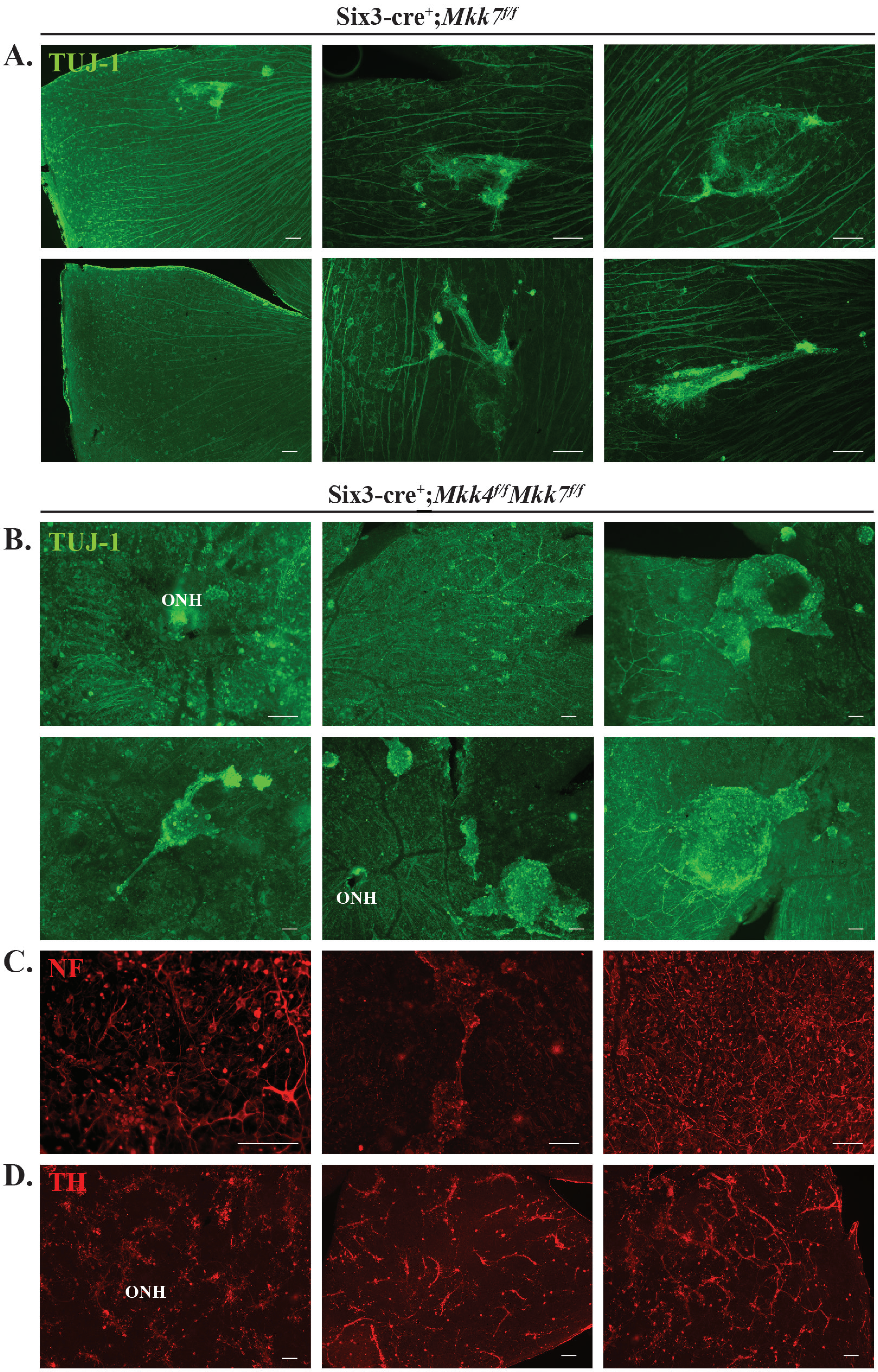

Supplement: Supplementary file 1 — Supplemental Material [file 41419_2018_1079_MOESM1_ESM.pdf]
